# Influence of light and anoxia on chemiosmotic energy conservation in *Dinoroseobacter shibae*

**DOI:** 10.1111/j.1758-2229.2010.00199.x

**Published:** 2011-02

**Authors:** Johannes Holert, Sarah Hahnke, Heribert Cypionka

**Affiliations:** Institute for Chemistry and Biology of the Marine Environment, University of OldenburgCarl-von-Ossietzky-Straße 9-11, D-26111 Oldenburg, Germany

## Abstract

In the present study we have investigated the influence of light and anoxia on the energetic state of the aerobic anoxygenic phototroph (AAP) *Dinoroseobacter shibae*. Respiration, chemiosmotic proton translocation and the adenylate energy charge (AEC) of the cells were measured comparing light versus dark and oxic versus anoxic conditions. Light caused a decrease of the respiration rates of washed cells. This might be a substitution rather than a direct inhibitory effect, because both photosynthesis and respiration contribute to the proton-motive force. As known from other AAPs, light alone did not induce proton translocation if applied to anoxic cell suspensions. However, additions of small oxygen pulses to anoxic cell suspensions caused two times more proton translocation in the light than in the dark. The AEC of the cells was measured by means of a modified luciferin-luciferase method. Growing cells of *D. shibae* kept an AEC of 0.93, indicating that the adenylate pool was highly phosphorylated. After harvesting and storing the cells under anoxic conditions for 2 h, the AEC dropped to 0.12. However, the cells remained reactive. Upon addition of oxygen, the AEC increased to its original value within 40 s by the formation of about 12 mM of intracellular ATP. There were no differences whether this recovery experiment was carried out in the dark or in the light. We conclude that *D. shibae* is able to change its energetic state not only in response to the light regime but also during oxic–anoxic transitions. Both responses appear suited to save *in situ* organic substrates and endogenous electron donors, thus enhancing the role of photosynthetic energy conservation.

## Introduction

Aerobic anoxygenic phototrophs (AAPs) are bacteria that carry out anoxygenic photosynthesis under oxic conditions. AAPs are not autotrophic and appear to use light energy as an additional, but never as their sole energy source (Shiba *et al*., 1979; Harashima *et al*., 1987). They form photopigments under suitable conditions only (Yurkov and Beatty, 1998; Kolber *et al*., 2001). Although AAPs harbour the complete set of photosynthesis genes, the pigment contents are lower than in anaerobic purple bacteria. Light-stimulated ATP formation (Shiba, 1984) and light-driven proton translocation (Okamura *et al*., 1986) were observed in preliminary experiments, but have not yet been studied in detail. There are several differences to the anoxygenic phototrophs with respect to the regulation patterns. While in the anaerobic phototrophs the expression of photosynthesis genes is repressed by molecular oxygen, the AAPs form bacteriochlorophyll only under oxic conditions (Harashima *et al*., 1980). Furthermore, bacteriochlorophyll synthesis occurs in the dark, and is inhibited by light (Harashima *et al*., 1980; Yurkov and van Gemerden, 1993). Thus, AAPs require the diel dark–night cycle to retain their photosynthetic apparatus. Cells grown under continuous illumination stay more or less colourless and are unable to use light energy.

Although AAPs are not primary producers, they might be of global significance in the oceans by slowing down cycling of organic carbon (Kolber *et al*., 2001; Beja *et al*., 2002). Their phototrophic activity will prevent organic matter from being remineralized and channel it into biomass instead. This function is comparable to the light-driven proton pump of proteorhodopsin-containing marine bacteria that are also heterotrophs and use light as an additional energy source (Beja *et al*., 2001; Bielawski *et al*., 2004).

Several studies on the global distribution of AAPs have shown that they are abundant mainly in marine habitats, but their occurrence varies with the environmental parameters (Kolber *et al*., 2001; Beja *et al*., 2002; Goericke, 2002; Allgaier *et al*., 2003; Gich *et al*., 2005; Schwalbach and Fuhrman, 2005; Cottrell *et al*., 2006; Lami *et al*., 2007; Rathgeber *et al*., 2008; Salka *et al*., 2008).

Beyond the biogeographical studies, physiological experiments are required to estimate how much aerobic anoxygenic photosynthesis contributes to the global biogeochemical cycles. However, several circumstances impede the design of those experiments. As described above, cultivation of AAPs requires light–dark cycles to enable them to form pigments in the dark and use them in the light. Second, although the photosynthetic reactions in AAPs do not consume or produce oxygen, the presence of oxygen is relevant not only for bacteriochlorophyll synthesis. If bacteriochlorophyll-containing cells are incubated under anoxic conditions they do not use light energy. The reason for this appears to be the fact that under anoxic conditions the primary electron acceptor is reduced and cannot accept electrons released from the reaction centre (Okamura *et al*., 1985; Garcia *et al*., 1994; Schwarze *et al*., 2000; Beatty, 2002). Third, in the laboratory it is difficult to keep the cells under the severe substrate limitation AAPs are exposed to in nature. This might be the reason that in many studies additional energy yield in the light was rather small (Harashima *et al*., 1987; Yurkov and van Gemerden, 1993).

The AAP studied here, *Dinoroseobacter shibae* strain DFL12^T^, was isolated from a marine dinoflagellate (Biebl *et al*., 2005). In well-designed experiments, Biebl and Wagner-Döbler (2006) studied nutrient deficiency in the absence or presence of light by exposing continuous cultures to interruption of medium supply. The cultures exhibited a base level of bacteriochlorophyll *a*, which decreased reversibly in the light and increased significantly during starvation in the dark to reach an increased level 16 h after medium supply was resumed. This indicated a slow regulatory response towards periodic starvation. Under simultaneous illumination and starvation conditions, these two effects apparently cancelled out each other, resulting in a steady-state level of bacteriochlorophyll *a*.

In the present study we carried out short-term experiments targeting the responses of *D. shibae* to light–dark and oxic–anoxic transitions. The latter were of special interest as the dinoflagellate host of *D. shibae* is often found in sand, sediments or in biofilms on larger organisms. Thus, it will be occasionally exposed to anoxic conditions. Correspondingly, the genome analysis of *D. shibae* (Wagner-Döbler *et al*., 2009) has confirmed the presence of several pathways involved in anaerobic respiration or fermentation. Our experiments show that light and oxic–anoxic transitions cause rapid and dramatic changes in the energetic state of the cells. The results are discussed with respect to their physiological and environmental relevance.

## Results and discussion

### Rapid changes of the energy charge during oxic–anoxic transitions

Growing cells and those harvested from the late exponential growth phase and kept under oxic conditions had an almost fully phosphorylated adenylate pool with an adenylate energy charge (AEC) value of 0.93. The average amount of ATP under these conditions was 53.4 nmol (mg protein)^−1^ and the amounts of ADP and AMP were 11.1% and 2.3% of that value respectively.

When the cells were stored anoxically in the dark at room temperature, the AEC dropped to 0.12 within 2 h. The adenylate pool consisted mainly of AMP under these conditions, while the amount of ADP did not change significantly and the ATP content decreased drastically to about 4% of the value under oxic conditions. The sum of the concentrations of the three adenylates remained constant.

**Fig. 1:**
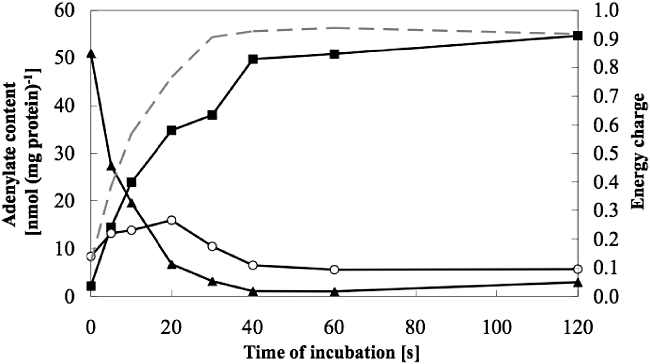
Transformation of adenylate nucleotides and adenylate energy charge (AEC) in washed, pigmented cell suspensions (see Supporting information S2 for more detail) of *Dinoroseobacter shibae* (OD_436_ = 10). To measure the AEC, a modified luciferin-luciferase assay was used which allows a precise quantification of ATP, ADP and AMP in a single assay (see Supporting information S5 for more detail). Cells were pre-incubated under anoxic conditions in the dark for 2 h. Upon addition of oxygen, fast ATP (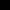) formation occurred. The AMP (▴) concentration decreased correspondingly, while ADP (○) increased only transiently. This results in an increase of the energy charge (dashed line). All values are averages from two independent measurements.

Upon subsequent addition of oxygen, the composition of the adenylate pool changed dramatically: within 40 s, the intracellular ATP concentration increased 23-fold to its previous value (Fig. 1). The AMP content decreased correspondingly, while the ADP concentration changed only transiently in the beginning of the experiment. After 40 s the concentrations of all three adenylates remained constant. Estimating the volume of the cytoplasm of a cell to be about 0.295 · 10^−15^ l assuming an average cell size of 1 µm in length and 0.7 µm in width and a cylindrical cell form with spherical endings (Biebl *et al*., 2005) it was calculated that the ATP concentration increased from 0.6 to 12.9 mM when oxygen was supplied.

Additional supply of light or succinate during the incubation with oxygen did not change the ATP formation rates and yields indicating that the ATP generation was running at maximum rate. Control experiments with unpigmented cells confirmed that light had no additive influence on the ATP generation rate or yield. Illumination of anoxically incubated cell suspensions without addition of oxygen did not result in an increase of ATP or ADP. Anoxically incubated cell suspensions treated with the protonophor TCS (3,3′,4′,5-tetrachlorosalicylanilide) were unable to generate ATP upon the addition of oxygen, indicating that this rapid ATP generation is based on chemiosmotic processes.

The intracellular concentrations of inorganic phosphate varied between 90 and 180 nmol per mg protein in different cell charges, but were always sufficient to explain the observed ATP formation. The orthophosphate decrease was nearly stoichiometric to the amount of ATP formed. However, phosphate consumption appeared to start when the ATP replenishment was finished already. This might hint on additional processes that are still to be unravelled.

Currently it is unknown whether the dramatic changes of the energetic state of the cells are a regulatory adaptation in *D. shibae*. As explained above, AAPs need the day-and-night rhythm to use light energy in a sustainable manner. There must be a regulation pattern that switches on chlorophyll synthesis in the dark. It would make sense to slow down other metabolic activities at the same time. Particularly, intracellular reserve material could be saved instead of oxidizing it in the dark. While for *Escherichia coli* it was reported that dropping of the AEC below values of 0.5 caused death of the cells (Chapman and Atkinson, 1977), the ability to reversibly decrease their energy charge to even lower values was shown for several other bacteria (Barrette *et al*., 1988). At such a low energy state, the metabolic activity of bacteria is usually strongly decreased. The activity of many enzymes involved in anabolic processes is lowered and protein synthesis is repressed, whereas the activity of enzymes contributing to the regeneration of ATP is increased (Atkinson, 1968).

### Respiratory and light-driven proton translocation

**Fig. 2:**
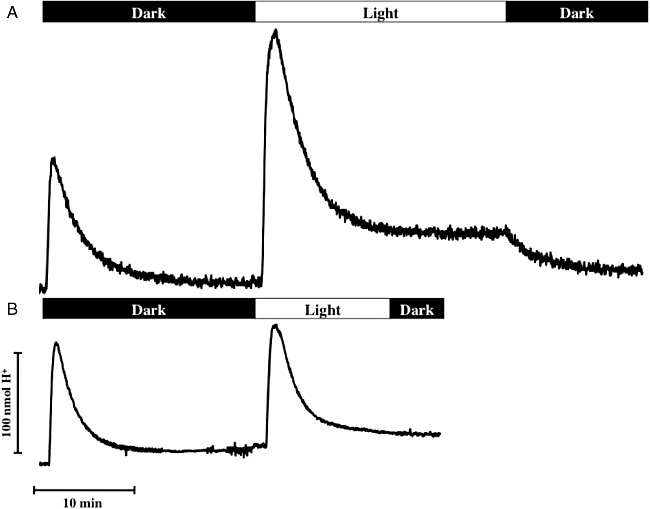
Oxygen- and light-induced proton translocation in washed, pigmented (A, OD_436_ = 9.6) and unpigmented (B, OD_436_ = 5.7) cell suspensions (see Supporting information S2 for more detail) of *Dinoroseobacter shibae* incubated under anoxic conditions (see Supporting information S4 for more detail). The first peaks result from the addition of 8 nmol of oxygen in the dark, the second peaks from the addition of 8 nmol of oxygen in the light (average intensity: 420 µE m^−2^ s^−1^, see Supporting information S1 for more detail).

While we did not detect a light effect on the rapid changes of the AEC upon oxygen additions, light utilization could be demonstrated in proton translocation and respiration experiments. The addition of small amounts of oxygen to dense cell suspensions incubated anoxically in non-buffered salt solution caused a transient decrease of the extracellular pH (Fig. 2). During the oxygen uptake phase (a period of about 2 min ending at the H^+^ maximum concentration) vectorial proton translocation and proton uptake proceeded concomitantly. After oxygen depletion, uptake of protons with typical first-order kinetics remained visible for several minutes. The latter process was slowed down by the presence of KSCN in the assay (Scholes and Mitchell, 1971). Maximum values of about 10 protons translocated per added oxygen atom (H^+^/O) were determined in the dark. This high value can be explained by the fact that an excess of reducing power was built up during the anoxic incubation.

If oxygen and light were supplied simultaneously, the amount of extruded protons was twice as high as in the dark (Fig. 2), demonstrating light-driven proton translocation. Illumination of anoxic suspensions without prior addition of oxygen did not result in any proton translocation as found with other AAPs before (Schwarze *et al*., 2000; Beatty, 2002). However, when oxygen was added to illuminated cells and illumination was maintained even after oxygen was depleted, light-driven proton translocation continued also under anoxic conditions. A steady state between light-driven proton extrusion and (light-independent) uptake was maintained until light was switched off (Fig. 2). Respiratory and light-driven proton translocation were inhibited completely by the uncoupler TCS. Unpigmented cells did not show light-induced proton translocation (Fig. 2).

Thus, light utilization as chemiosmotic energy source was demonstrated. Our findings are in accordance with an estimation of Beatty (2002), who calculated an extent of 10–50% of the total energy generation in AAPs to be light-driven. The finding that light-driven energy conservation in *D. shibae* continued under anoxic conditions if it was started by an oxygen pulse in the light is new. In previous studies on *Roseobacter denitrificans* (Okamura *et al*., 1985; Garcia *et al*., 1994) it was concluded that a high redox potential of the primary electron acceptor Q_A_ is responsible for the inability to transfer electrons under anoxic and completely reduced conditions. In contrast, Schwarze and colleagues (2000) reported a much lower redox potential for Q_A_ in *R. denitrificans* and held an over-reduction of the ubiquinone pool under anoxic conditions responsible for the inhibition of cyclic electron transport. Our observations do not differentiate between these two possibilities, but exclude that the presence of oxygen is obligate for photosynthetic light utilization in *D. shibae*.

### Light partially substitutes oxygen respiration

Respiration was measured with washed cell suspensions taken from dark-grown overnight cultures and stored anoxically on ice. The endogenous oxygen uptake rate of these cells was on average 26.6 nmol O_2_ per minute and mg protein in the dark (Table 1). If the suspension was illuminated (1500 µE m^−2^ s^−1^) the endogenous rate decreased reversibly by 34%. When succinate (as the growth substrate) was supplied as electron donor, the oxygen uptake rate was 43.5 nmol O_2_ per minute and mg protein, and decreased by 24% under illumination (Table 1, see Supporting information S3 for more detail). The addition of other organic substrates resulted in lower respiration rates than with succinate. This indicated that light-driven electron transport was operative and could replace electron transport to oxygen (Harashima *et al*., 1989), thus saving both endogenous and exogenous electron donors. Cells grown under continuous light had no pigments and did not show light-sensitive respiration.

**Table 1:** Aerobic respiration rates of washed, pigmented cell suspensions (see Supporting information S2 for more detail) of *D. shibae* in Hepes buffer (10 mM, pH 7.75, supplemented with NaCl 20 g l^−1^, KCl 0.5 g l^−1^) measured with a Clark-type oxygen electrode (Bachofer, Reutlingen, Germany).

	Condition		
	
Respiration rates [nmol O_2_ min^−1^ (mg protein)^−1^] Electron donor	Dark	Light	Dark again
Endogenous	26.6 ± 6.9	17.6 ± 5.6	20.7 ± 6.72
Succinate	43.5 ± 9.8	33.2 ± 6.1	40.7 ± 9.4

The influence of light (average intensity 1500 µE m^−2^ s^−1^, see Supporting information S1 for more detail) on endogenous and succinate-stimulated oxygen consumption rates was measured. The data represent the average of three independent measurements.

A decrease of the oxygen consumption rate as found with *D. shibae* in the light was described for AAPs already by Harashima and colleagues (1982). Later biochemical investigations revealed that light-driven and respiratory electron transport in AAPs are partially mediated via identical electron carrier systems like quinones and cytochromes (Yurkov and Beatty, 1998; Schwarze *et al*., 2000; Candela *et al*., 2001), which therefore function as a bottleneck of the electron transport (Beatty, 2002). That in *D. shibae* similar mechanisms may be involved is supported by genetic evidence regarding the composition of the electron transport systems, which is similar to that of mitochondria (Wagner-Döbler *et al*., 2009).

The observed 23-fold increase of the ATP level (or the formation of 12 mM intracellular ATP) within 40 s raises the question whether such rapid ATP formation can be explained by the observed respiration rates and proton translocation. Assuming the cell size calculated above, the production of 12 mM ATP means that each cell generated about 2.1 · 10^6^ molecules within 40 s. The maximum respiration rate of *D. shibae* was about 1.4 · 10^6^ molecules O_2_ per cell and minute. Coupled to proton translocation with an H^+^/O ratio of 10 a number of 2.8 · 10^7^ protons could be translocated per minute. Assuming that the ATP synthase requires four protons per ATP, 7 · 10^6^ ATP molecules could be formed per minute, which is sufficient to explain the observed ATP generation in *D. shibae*. From other bacteria even higher turnover rates of the ATP pool of less than once per second have been reported (Holms *et al*., 1972; Karl and Bossard, 1985; Norling *et al*., 1989). Okamura and colleagues (1986) analysed the chemiosmotically driven ATP formation in chromatophores of the AAP *R. denitrificans* supplied with ADP and inorganic phosphate. They found ATP generation rates which were about a factor of 10 smaller than those observed here with *D. shibae*.

### Environmental relevance

In the present study we have shown that *D. shibae* rapidly adjusts its energetic state to the oxygen regimen: when shifted from oxic to anoxic conditions, the energy charge was drastically but reversibly lowered. Furthermore, it was proven that light can contribute a major part to the chemiosmotic energy conservation under appropriate conditions. Light utilization is suited to save *in situ* organic substrates and endogenous electron donors, thus promoting assimilation instead of reoxidation of organic compounds. With respect to the variable abundances of AAPs found in different regions of the oceans one should regard that the special abilities of AAPs do not provide a competitive advantage everywhere. In strictly oligotrophic areas of the oceans, iron, phosphorous or nitrogen will be growth-limiting for all organisms, while a relatively large fraction of organic compounds is released by primary producers. Similarly, in coastal zones organic substrates are often not the limiting factor. The special ecological niche for AAP is given in euphotic habitats where electron donors are controlling secondary production.

## Supporting information

**Supporting information S1.** Illumination and light measurement.

**Supporting information S2.** Cultivation and pigment analysis.

**Supporting information S3.** Influence of light on the respiration rate of *Dinoroseobacter shibae*.

**Supporting information S4.** Measurement of chemiosmotic proton translocation.

**Supporting information S5.** Measurement of energy charge.
