# Supplementary material for: Influence of light and anoxia on chemiosmotic energy conservation in Dinoroseobacter shibae
Source: Environ Microbiol Rep. 2011 Feb;3(1):136–41. doi: 10.1111/j.1758-2229.2010.00199.x (PMC3064978; doi:10.1111/j.1758-2229.2010.00199.x)
Supplement: Supplementary file 1 [file emi40003-0136-SD1.rtf]

Supporting information

Influence of light and anoxia on 
chemiosmotic energy conservation in Dinoroseobacter shibae

Johannes Holert1), Sarah Hahnke and Heribert Cypionka*)

Institute for Chemistry and Biology of the Marine Environment, University of Oldenburg, 
Carl-von-Ossietzky-Straße 9-11, D-26111 Oldenburg, Germany
1)Present Address: University of Konstanz, Dept. of Biology, Microbial Ecology, Universitätsstr. 10
D-78457 Konstanz
*) For correspondence. E-mail: Cypionka@icbm.de; Tel. (+49) 441 798 5360; Fax (+49) 441 798 3404

S1: A halogen lamp (KL 1500 electronic, Schott, Mainz, Germany) was used for illumination. Light intensity was measured with a LICOR Li189 radiometer (Lincoln, NE  68504 
USA, equipped with a quantum sensor) and was calculated according to Overmann et al. (1992).

References: 

Overmann, J., Cypionka, H., and Pfennig, N. (1992) An extremely low-light-adapted phototrophic sulfur bacterium from the Black Sea. Limnol Oceanogr 37: 150-155.


S2: Pigmented cultures were grown in the dark, while control cultures without pigments were grown under constant illumination (Osram, Concentra 60 W). The in vivo content of the photopigments was analyzed by recording the absorption spectrum of whole cells in a UV/VIS spectrophotometer (Perkin Elmer, Lambda 2S) with a resolution of 1 nm from 950 to 350 nm. Peaks at 805 nm and 870 nm indicate the presence of pigments. Cultures without peaks at these wavelengths and without red coloration are regarded as unpigmented.

S3: Influence of light on the respiration rate of Dinoroseobacter shibae. Oxygen consumption of a washed, pigmented cell suspension in HEPES buffer (10 mM, pH 7.75, supplemented with NaCl 20 g  liter1, KCl 0.5 g  liter1) in the presence of 10 mM succinate measured with a Clark-type oxygen electrode (Bachofer, Reutlingen, Germany). When light (1500 µE m-2 sec-1) was switched on, the respiration rate was reversibly lowered. Respiration rates were calculated from regression analysis (dashed lines). 


S4: Vectorial proton translocation was analysed via pH measurements (Inlab®423 electrode, Mettler Toledo) by addition of small oxygen pulses or light to non-buffered cells suspensions (Mitchell and Moyle, 1967). Cells were suspended in NaCl (20 g  l1) with KCl (0.5 g  l1) and KSCN (50 mM). Thiocyanate is a permeable anion that slows down the reflux of translocated protons by lowering the membrane potential (Mitchell and Moyle, 1967; Fitz and Cypionka, 1989 and 1991). The pH of the solution was adjusted to 7.4 with anoxic KOH or HCl (10 mM). To calibrate the system, known amounts (5 to 40 nmol) of anoxic HCl (1 mM) were added and the resulting pH changes were recorded. Data acquisition was performed via a 12 bit A/D converter with the custom made program MPWin (http://www.pmbio.icbm.de/download/MPwinU12.zip).
The experiment was started by addition of a small amount of oxygen-saturated KCl to the cell suspension, resulting in a short phase of respiration and proton translocation (plus concomitant proton uptake), followed by a phase of uptake of protons via the ATPase after oxygen depletion. For calculation of the maximum amount of translocated protons, the second part of the pH curve was extrapolated to the time when oxygen was added, assuming first-order kinetics of proton uptake.

References: 

Mitchell, P., and Moyle, J. (1967) Respiration-driven proton translocation in rat liver mitochondria. Biochem J 105: 1147-1162.

Fitz, R.M., and Cypionka, H. (1989) A Study on electron transport-driven proton translocation in Desulfovibrio desulfuricans. Arch Microbiol 152: 369-376.

Fitz, R.M., and Cypionka, H. (1991) Generation of a proton gradient in Desulfovibrio vulgaris. Arch Microbiol 155: 444-448.


S5: Measurement of energy charge
Experiment and extraction of adenylates
Washed cell suspensions (0.5 ml with a known number of cells between 109 and 1010) were incubated in 3 ml Hungate tubes sealed with butyl rubber stoppers in the dark and flushed with nitrogen at room temperature. Thereafter different conditions were applied with respect to the availability of oxygen, light and organic substrates, before the cells were extracted with perchloric acid or used for orthophophate analysis by the molybdenum blue method (Holman, 1943). To immediately stop any further reactions, 500 µl of ice-cold perchloric acid (3 M) were injected into the cell suspension. Under temporary shaking the nucleotides were extracted for 30 minutes on ice. After centrifugation (4 °C, 13,000 rpm, 4 min) the supernatant was neutralized with KOH (3 M) and kept on ice to enable precipitation of KClO4. The samples were neutralized by 250 µl Tes (N-tris[hydroxymethyl]methyl-2-aminoethansulfonic acid) buffer (10 mM, pH = 7.75) and centrifuged again. The supernatant was carefully transferred into new reaction tubes and kept on ice (or instantly frozen) for later adenylate analysis. 

One-cuvette measurement of ATP, ADP and AMP
To measure the energy charge, a modified luciferin-luciferase assay was developed which allows a precise quantification of ATP, ADP and AMP in a single assay. As the luciferase detects only ATP, the conventional analysis of ADP and AMP was done in separate assays after stepwise transformation of ADP and AMP to ATP (Kimmich et al., 1975). The fact that the ADP and AMP concentrations are often small and appear only as small offset to the bulk ATP gives rise to errors. This is even worse as the luciferase is sensitive to small chemical and even physical (movement and temperature) variations in the assays (Kahru and Vilu, 1990). We combined these three measurements into one, thereby eliminating major sources of error. After recording the luminescence of ATP, pyruvate kinase and later adenylate kinase were added to the reaction mixture, and the transformation of ADP and AMP into ATP was recorded in real time.
The ATP sensitive assay LBRS010 from Biaffin GmbH (Kassel, Germany) was used. Light emission was recorded using a LKB Wallac 1250 luminometer. The reaction mixture, containing 20 µl phosphoenol pyruvate solution (1 mM), 10 µl MgSO4 solution (50 mM) and 20 µl of the luciferinluciferase reagent was added to the cuvette and a blank signal was recorded. To start the measurement of ATP, 20 µl of cell extract were added and the light emission was recorded. To determine ADP and AMP 4 µl pyruvate kinase (MP Biomedicals; 1 U/µl in HEPES 50 mM, pH 7.75) and 3 µl of adenylate kinase solution (Sigma; 1 U/µl in HEPES 50 mM, pH 7.75) were added to the reaction mix. The formation of ATP was recorded until a stable signal was achieved. Internal calibration with ATP standard solution was performed at least twice after every step. Curves showing a drift were extrapolated to the time of enzyme addition and the maximum luminescence was used for proper calculation. Recovery rates were determined from standard mixtures containing different ratios of all three adenylate nucleotides, which were equally treated as cell suspensions. The modified luciferinluciferase method allowed the detection of ATP down to 0.1 pmol as described in the manufacturers handbook while the detection limit for AMP was about 1 pmol. 

References: 

Holman, W.I. (1943) A new technique for the determination of phosphorus by the molybdenum blue method. Biochem J 37: 256-259.

Kimmich, G.A., Randles, J., and Brand, J.S. (1975) Assay of picomole amounts of ATP, ADP, and AMP using the luciferase enzyme system. Anal Biochem 69: 187-206.

Kahru, A., and Vilu, R. (1990) Role of adenine nucleotides in the regulation of bacterial energy metabolism: theoretical problems and experimental pitfalls. Microbios 62: 83-92.
